# Living With Ambiguity: A Metasynthesis of Qualitative Research on Mild Cognitive Impairment

**DOI:** 10.1093/geront/gnv067

**Published:** 2015-08-27

**Authors:** Tim Gomersall, Arlene Astell, Louise Nygård, Andrew Sixsmith, Alex Mihailidis, Amy Hwang

**Affiliations:** ^1^School of Health & Related Research, University of Sheffield, Sheffield, UK.; ^2^Department of Neurobiology, Care Sciences, and Society, Karolinska Institutet, Stockholm, Sweden.; ^3^Department of Gerontology, Simon Fraser University, Vancouver, Canada.; ^4^Toronto Rehabilitation Institute, University of Toronto, Toronto, Canada.

**Keywords:** Dementia, Attitudes and perception toward aging/aged, Cognition, Ethics (research, practice, policy, individual choices), Qualitative research methods, Sociology of aging/social gerontology, Well-being

## Abstract

**Purpose of the study:**

Mild Cognitive Impairment (MCI) is a diagnosis proposed to describe an intermediate state between normal cognitive aging and dementia. MCI has been criticised for its conceptual fuzziness, its ambiguous relationship to dementia, and the tension it creates between medical and sociological understandings of “normal aging”.

**Design and Methods:**

We examined the published qualitative literature on experiences of being diagnosed and living with MCI using metasynthesis as the methodological framework.

**Results:**

Two overarching conceptual themes were developed. The first, MCI and myself-in-time, showed that a diagnosis of MCI could profoundly affect a person’s understanding of their place in the world. This impact appears to be mediated by multiple factors including a person’s social support networks, which daily activities are affected, and subjective interpretations of the meaning of MCI. The second theme, Living with Ambiguity, describes the difficulties people experienced in making sense of their diagnosis. Uncertainty arose, in part, from lack of clarity and consistency in the information received by people with MCI, including whether they are even told MCI is the diagnosis.

**Implications:**

We conclude by suggesting an ethical tension is always at play when a MCI diagnosis is made. Specifically, earlier support and services afforded by a diagnosis may come at the expense of a person’s anxiety about the future, with continued uncertainty about how his or her concerns and needs can be addressed.

Mild cognitive impairment (MCI) is a relatively new diagnostic category that first appeared in the medical literature in the 1990s (Flicker, Ferris, & Reisberg, 1991; Petersen et al., 1997). MCI was proposed as “a boundary or transitional state between normal aging and dementia” (Petersen et al., 1999, p. 303) although not everyone who is diagnosed with MCI goes on to develop dementia (Sachdev et al., 2013). MCI is generally defined by memory performance below the normal range for a person’s age, alongside preserved functioning in daily activities. The problems are noticeable to the person themselves and to close others; however, these problems do not significantly impact daily functioning (Chertkow et al., 2008; Petersen, 2004). Petersen (2004) classified MCI types based on memory deficits: amnestic type MCI refers to impaired memory with (multiple domain) or without (single domain) other cognitive deficits; nonamnestic type MCI refers to impairment in one or more cognitive area(s) (i.e., single or multiple domains) other than memory. Petersen (2004) indicated that these different variations of MCI have different levels of risk for advancing to Alzheimer’s or other types of dementia, with amnestic MCI increasing the risk of Alzheimer’s disease and vascular dementia, whereas the nonamnestic types of MCI were linked to development of other dementia subtypes (e.g., fronto-temporal dementia and dementia with Lewy bodies). However, others have found that these distinctions in type of MCI do not so accurately predict types of dementia (Fischer et al., 2007). Additionally, it has been suggested that complex activities of daily life may be affected (e.g., Binegar, Hynan, Lacritz, Weiner, & Cullum, 2009; Kim et al. 2009; Nygård, 2003; Nygård, Pantzar, Uppgard, & Kottorp, 2012).

In attempt to resolve this uncertainty, the U.S. National Institute on Aging and the Alzheimer’s Association convened a working group to develop criteria for identifying “the symptomatic predementia phase of Alzheimer’s disease” (p1, Albert et al., 2011). This working group proposed two sets of criteria: one set of “core clinical criteria” that could be implemented by health care providers without access to brain imaging and other resources, and a second set for researchers with access to imaging and other diagnostic technologies. The working group’s proposed “core clinical criteria” are almost identical to those proposed by Petersen and colleagues (1999): concern regarding a change in cognition; objective impairment in one or more cognitive domain; preservation of independence in functional abilities; and not meeting criteria for dementia (Albert et al., 2011). Although this may have helped address the uncertainty by restating the original Petersen criteria, the fifth edition of the Diagnostic and Statistical Manual (DSM-V; American Psychiatric Association, 2013), arguably complicates matters further by not using the term MCI at all. Instead, the DSM-V contains the diagnostic criteria for mild neurocognitive disorder. These are similar to the core clinical criteria from Albert and colleagues (2011): evidence of cognitive decline based on informant concerns and objective testing, which is not sufficiently severe to interfere with independence. Additionally, the DSM specifies that the cognitive deficits do not occur exclusively in the context of a delirium and are not primarily attributable to another mental disorder (e.g., major depressive disorder, schizophrenia).

Given these inconsistencies, it is challenging to clarify how many people may be living with MCI. A recent systematic review (Ward, Arrighi, Michels, & Cedarbaum, 2012) found a median MCI prevalence, derived from 10 population-based studies, of 26.4%. However, individual estimates varied widely, from 3% to 42%. The study reporting the highest prevalence (Artero et al., 2008) was among the largest of the reviewed studies, including 6,892 French community-dwelling people aged 65 years and over. These variations may be one consequence of the lack of agreement over diagnostic criteria. Additionally, cross-cultural differences in help-seeking may have played into the observed cross-national discrepancies. Although this lack of consensus is frustrating and unhelpful from both clinical and research perspectives, philosophical and ethical controversies have also arisen (Sabat, 2006; Werner & Korczyn, 2008).

Whether from a clinical or patient perspective, the significance of a physician’s choice of label can hardly be overstated. The act of diagnosis has far-reaching implications for a person’s sense of self, especially if the diagnosis relates to mental health (Gergen, Hoffman, & Anderson, 1996). However, the implications for the individual (and their family) of being given a diagnosis of MCI and sent home to live with the label—particularly in terms of prognosis—are not well understood. MCI does not yet loom large in the collective consciousness, and this may have important repercussions in terms of personal experience and identity. As MCI is increasingly taken up in clinical practice, it is important to take stock of how people make sense of the label, and what the implications of being diagnosed might be. Hence, we aimed to synthesise the existing qualitative literature on people’s experiences of being diagnosed, and living with, a MCI diagnosis.

## Method

### Searches

Searches were undertaken across four key bibliographic databases: PsycINFO, CINAHL, MEDLINE, and Web of Science. As MCI is a recent category, searches were limited from the year 1998 [the year prior to the Petersen and colleagues (1999) publication], with the latest search conducted in August 2014. MCI-related terms were combined with terms for qualitative methods, and supplemented by manual searches of Google Scholar and the reference lists of included studies. Records were downloaded into Reference Manager® software, and sifted in a two-stage process. First, titles and abstracts were screened for relevance, and any articles clearly not meeting the inclusion criteria (Table 1) were excluded. Then, the remaining full-text articles were examined for inclusion.

**Table 1.** Inclusion/Exclusion Criteria

Inclusion
Qualitative studies, or mixed-method studies which presented substantial qualitative data
E.g., Grounded theory, thematic analysis, phenomenology, discourse analysis, etc.
Explicitly addresses experiences of being diagnosed/ living with MCI
Exclusion
Quantitative studies/ mixed-method studies lacking substantial qualitative data
Conference abstracts
Reviews
Studies exclusively of participants with dementia
Cognitive impairment secondary to another illness or treatment (e.g., Parkinson’s, diabetes, chemotherapy)
Does not address MCI experiences

### Analysis

There are many approaches to qualitative metasynthesis research, with researchers placing varying levels of emphasis on different aspects of the process (Barnett-Page & Thomas, 2009; Thorne, Jensen, Kearney, Noblit, & Sandelowski, 2004). Although the conceptual boundaries between different labels are fuzzy, the approach taken in this study most closely resembles that described in the classic monograph from Noblit and Hare (1988), and developed by Britten and colleagues (2002), Campbell and colleagues (2003), and Gomersall, Madill, and Summers (2011). Like these researchers, our methodological starting point was Turner’s (1980) theory of social explanation, according to which understanding of social phenomena proceeds through comparison—examining how findings from individual studies are similar or different, and seeking to understand the reasons for differences to develop theory that goes beyond the immediate research contexts. In terms of the specific types of synthesis outlined by Noblit and Hare, this article includes both *reciprocal translational analysis* and *lines of argument* synthesis. For example, despite wide variations in the definition of MCI in individual studies, ambiguity surrounding MCI and the confusion this caused participants was ubiquitous throughout the different research contexts, and we sought to capture this in the theme, *living with ambiguity.* However, the implications of an MCI diagnosis on participants’ sense of self were heterogeneous, and the way this manifested appeared to depend, to a certain extent, on the methodologies of individual studies themselves. For example, studies examining participant biographies threw perceptions of the past into sharper relief, whereas studies focusing on participants’ engagement in everyday activities tended to emphasise the present more. Additionally, as we go on to suggest, how the diagnosis of MCI was actually delivered to participants remains a “black box” which, if more data had been available, may have thrown light on why different people were making sense of MCI in often quite different ways.

The specific synthesis procedures were as follows. After deciding on our topic and identifying data through systematic database searches, the lead author engaged in an iterative reading of the literature to become well acquainted with the concepts within each article. Data extraction tables were created in Microsoft Word®, in which the lead author recorded details of study settings, participants, methodological approach, and the key themes/concepts from each individual study. In terms of these themes, the data extraction tables remained close to the original data (by drawing terms and quotations directly from the literature), and mindful of the context of each study, thereby retaining some of the “vitality, viscerality, and vicariism of the […] original studies” (Sandelowski, Docherty, & Emden, 1997, p. 366).

In the next stage, themes were examined across studies, in an attempt to establish a “dialogue” between them (Zimmer, 2006). Paper copies of individual data extraction tables were compared, and notes were made on similarities and discrepancies on the corresponding electronic copies. Much like a grounded theory analysis (Strauss & Corbin, 1997), this involved constant comparison, and seeking negative cases to refine emerging hypotheses. We began, through discussions among the research team, developing theories about how themes from different studies were related. Finally, the studies were translated by the lead author into more abstract, overarching concepts, which were again discussed among all authors. In so doing, we developed “third-order” interpretations: research participants interpreted the meaning of their experience in their talk (i.e., first-order interpretation), which was subsequently interpreted and selectively presented by researchers involved in the primary studies (second-order), and finally analysed for the synthesis. Hence, our interpretations were inevitably shaped by (a) the research material available to us, and (b) our own perspectives as researchers and practitioners. The lead author and A.A. have a background in psychology; L.N. is an occupational therapist, A.S. is a sociologist, and A.H. and A.M. are involved in software development. Consequently, we brought a wide range of concepts and ideas to our discussions of the analysis—especially psychological theory and research on the nature of the self, the importance of meaningful activities of daily living, the social and ethical implications of novel diagnostic entities, and the relevance of innovative technologies to supporting well-being in older adults. As Erwin, Brotherson, and Summers (2011) have argued, conducting a metasynthesis across a range of disciplines “can help bridge the gap between research and practice because of the representation of rich and diverse perspectives regarding a body of knowledge in the field” (p. 197). It is important also to acknowledge our research priorities so the reader has a clear sense of how they influenced the synthesis process, and why certain aspects of MCI experiences may have been emphasised more than others.

## Findings

### Study Details

**Figure 1.**
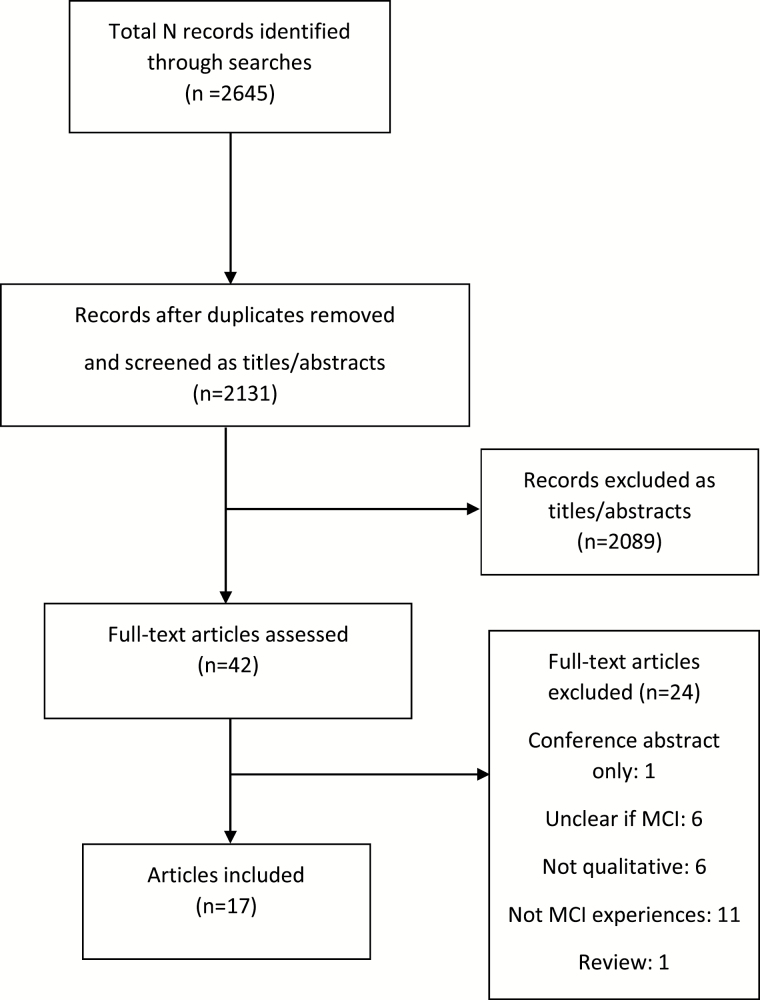
Study selection flow chart.

A total of 2,131 records were retrieved after duplicates were removed. Most (2,089) were excluded at title or abstract stage. Twenty-four full-text articles were removed, leaving a total of 17 for inclusion in the metasynthesis. The study selection process is summarised in Figure 1.

Studies were conducted in a variety of countries: the United States (10 studies), United Kingdom (3 studies), Sweden (2 studies), and the Netherlands, Canada, and Taiwan (1 study each). Generally, participants were recruited from specialist AD or memory clinics. Data collection methods predominantly comprised semistructured interviews, or focus groups. Two studies also incorporated “real-time” participant observations: Damianakis, Crete-Nishihata, Smith, Baecker, and Marziali (2010) observed participants’ viewings of multimedia biographies, whereas Rosenberg and Nygård (2013) observed participants’ uses of everyday technologies—such as cell phones, microwave ovens, and DVD players—and asked them to provide concurrent reflections and explanations on what they were doing. The analytical methods were usually described as grounded theory, though some studies described thematic analysis, or variants of phenomenology. One study (Saunders, de Medeiros, & Bartell, 2011) used discourse analysis. Several studies included participants with both MCI and AD, and a wide variety of MCI definitions were used as inclusion criteria. Further details are provided in Table 2.

**Table 2.** Study Characteristics

Background details				Methodology	
Authors, year	Country, setting	Participants	MCI definition for inclusion	Data collection method	Analytical strategy
Banningh and colleagues (2008)	Netherlands, Nijmegen University, 3 memory clinicians	*N*, total: 8	Petersen and colleagues (2001) criteria for amnestic MCI	60–75min protocol- based interviews, including: changes, thoughts or reflections, emotions, behaviour, social implications.	Grounded theory. Open coding independently by two researchers, followed by development of higher- order concepts.
		*N*, men: 1			
		Mean age (*SD*): 74.8 (8.1)			
Beard and Neary (2013)	United States. Research registry in a large Midwestern city	*N*, total: 18	Diagnosis of amnestic MCI within 3 years	In-depth interviews and focus groups. Non-standardized topic guide used for interviews. Respondents encouraged to lead the conversation.	Grounded theory. Open, line- by-line coding, followed by consolidation to identify key variables.
		*N*, men: 6			
		Mean age (*SD*): 76 (NR)			
Berg and colleagues (2013)	Sweden. The longitudinal Gothenburg MCI study	*N*, total: 17	Stable MCI for ≥7 years, based on medical history	Interviews (1–2hr), starting with open questions; followed with specific questions on cognitive problems, health, well-being, stress, relationships, and perspective on the future.	Thematic analysis. Transcripts read iteratively, then coded line-by-line. Clusters of themes developed for each transcript, and compared across transcripts to generate higher- order themes.
		*N*, men: 11			
		Mean age (*SD*): 72 (NR)			
Blieszner and colleagues (2007) ^a^	United States, 3 memory clinics in Virginia	*N*, total: 73 couples	Clinical MCI diagnosis using tests to rule out dementia and potential reversible causes of memory loss	Open-ended interviews separately with partners and PWMCI. Topics included: perception of memory problems, coping strategies, and views of future care options.	Interview transcript synopses created for each couple and coded. Salient constructs identified from each transcript, then grouped into categories related to “ambiguous loss” model. Analysis combined sensitising concept with inductive analytical strategies based on grounded theory.
		*N*, men: 73 (1/3 of PWMCI were female; 2/3 male)			
		Mean age (*SD*): PWMCI: 75 (6.09); partners: 71.2 (8.68)			
Blieszner and Roberto (2010)	United States, memory clinics in Virginia	*N*, total: 86	Petersen (2004) criteria by clinical assessment	Face-to-face structured interviews with open- ended questions	Open coding followed by focused coding. Triangulation with multiple team members
		*N*, men: 13			
		Mean age (*SD*): 66.9 (13.7)			
Corner and Bond (2006)	United Kingdom, local geriatric psychiatry service, memory clinic, local AS branch, and local day centres	*N*, total: 2 case studies (1 couple, 1 single)	NA—AD or no diagnosis. Accounts used “to gain a picture of the likely experiences and opinions of people with MCI”	Series of interviews with older people with early-stage dementia (*n* = 14), caregivers (*n* = 8), and older people neither diagnosed with, nor caring for someone with dementia (*n* = 15). Two case studies selected to illustrate the likely experience of MCI.	Grounded theory methods, including line-by-line coding and constant comparison.
		*N*, men: 1			
		Mean age (*SD*): NR			
Damianakis and colleagues (2010) ^a^	Canada, Ontario, geriatric care centre in a large urban setting	*N*, total: 12 (6 MCI, 6 AD)	NR	Participant multimedia biographies (MB) developed. Participants were shown MBs and responses were videotaped. Interviews also conducted at baseline, and 3- and 6-months. Open-ended questions examined: experience of developing and viewing the MB; and perceived impact of MB on communication/ relationships.	Content analysis. Manifest content extracted, and latent content (interpretations of participants’ meanings) developed. Two researchers independently performed line-by-line coding, then developed higher-order categories through comparisons. Nonverbal reactions to MBs were recorded and analyzed.
		*N*, men: 5 (4 MCI, 1 AD)			
		Mean age (*SD*): 79.6 (9.7)			
Davies and colleagues (2010) ^b^	United States, Stanford/ VA Alzheimer’s Research Centre	*N*, total: 23 (14 dementia, 9 MMI)	Petersen and colleagues (1999) criteria	Focus groups. Prompting questions pertaining to issues of intimacy/ sexuality.	“Indexing” thematic analysis. “reading the transcripts and extracting words or labels that related to the content […] repeated several times to allow for the emergence of new themes” (p. 620).
		*N*, men: 17 (10 dementia, 7 MMI)			
		Mean age (*SD*): 75 (8)			
Frank and colleagues (2006)	United States (2 memory clinics) and United Kingdom (1 memory clinic)	*N*, total: 67 (20 MCI patients/11 informants; 20 AD patients/16 informants)	Petersen and colleagues (2001) criteria	Two 90-min focus groups with PWMCI (*n* = 11), and two with AD (*n* = 16). Topics included impact of MCI/AD on everyday life, social relationships, experience of diagnosis, reaction to symptoms, and use of coping strategies.	Thematic analysis—interpretive summary created for each focus group. Analysis aims were: to organise responses by theme, to compare MCI and AD experiences, and compare patient and informant groups.
		*N*, men: 10 patients; 3 informants (MCI only)			
		Mean age (*SD*): patients: 72 (11); informants: 73 (8.5) (MCI group only)			
Kuo and Shyu (2010) ^b^	Taiwan—neurological clinic in Taoyuan	*N*, total: 10	NR	Semi-structured interviews 40–90min. Topics included changes in the partner, strategies for handling changes, and impact on feelings and family life.	Grounded theory. Line-by-line coding followed by constant comparison. Memos used to document emerging concepts.
		*N*, men: 1			
		Mean age (*SD*): 50.8 (11.42)			
Lingler and colleagues (2006)	United States, Pittsburgh Alzheimer Disease Research Centre	*N*, total: 12	Memory deficits (≥1.5 *SD* below mean), otherwise preserved cognition	Semi-structured interviews (45– 60min). Open-ended questions about impact of MCI.	Grounded theory. Line-by-line coding, then examine patterns and cluster codes. Emerging findings discussed and refined at team meetings.
		*N*, men: 6			
		Mean age (*SD*): 78 (5.6)			
Lu and Haase (2009) ^b^	United States, Alzheimer Disease Centre Clinic, Indiana	*N*, total: 11^c^	Clinical examination by a geriatric neurologist. Petersen and colleagues (1999) criteria	Open-ended, narrative interviews (45–90min). Open- ended question provided to the participant at least 7 days prior to interview asking about experience of diagnosis/ living with diagnosis.	Colaizzi’s phenomenology. Significant statements “identified, restated, and formulated into meanings by the first author.” These were discussed among the research team, and organised into thematic categories.
		*N*, men: 5			
		Mean age (*SD*): 72 (11.22)			
Roberto and colleagues (2011)	United States, memory clinics in 6 cities	*N*, total: 56 families	Clinically confirmed MCI [Petersen and colleagues (1999) criteria]	Semi-structured interviews with structured questionnaires at the beginning and end of the interview	Grounded theory. Open coding followed by organisation into higher-order codes, and constant comparison.
		*N*, men: 34			
		Mean age (*SD*): 76.5 (7.06)			
Roberto and colleagues (2013)	United States, memory clinics in 6 cities	*N*, total: 40 couples	Clinical diagnosis to rule out dementia and potential reversible causes of memory loss	3 consecutive interviews over 37.4 months, conducted separately with PWMCI and partners. Each round of interviewing followed up on changes experienced since the last interview.	Grounded theory. Open coding followed by organisation into higher-order codes, and constant comparison.
		*N*, men: 40			
		Mean age (*SD*): Carers: 71.1 (8.2); PWMCI: 74.6 (7.0)			
Roberts and Clare (2013)	North Wales: 4 specialist memory clinics	*N*, total: 25	Clinical diagnosis of MCI based on Petersen and colleagues (2001) criteria	Semi-structured interviews (11–30min). Topics included feelings that day, life changes since becoming older, and current situation / functioning	IPA. Iterative reading of interview transcripts, memo making, then analysis of clusters of themes. Themes were then compared across transcripts.
		*N*, men: 16			
		Mean age (*SD*): 76 (9.15)			
Rosenberg and Nygård (2013)	Sweden, Karolinska Institutet	*N*, total: 20 (10 MCI, 10 AD)	Petersen (2004) criteria	Semi-structured interviews, plus demonstration of an everyday technology in home environment, while explaining/ reflecting on actions.	Grounded theory. Initial open coding in Atlas TI software, then focused comparison of codes. Codes were compared between PWMCI and AD, and differences and similarities noted.
		*N*, men: 8			
		Mean age (*SD*): 68.2 (range: 56–87)			
Saunders and colleagues (2011)	United States, Georgetown University Neurology Department	*N*, total: 60 (31 with cognitive impairment; 29 without)	NR	Tape-recordings of routine neurological examinations. Transcribed using standard approach for discourse analysis.	Discourse analysis/mixed methods. Communicative coping behaviours sought, and coded occurrence of justifications/explanations of memory loss and health, and humour. Also included a statistical analysis of talk patterns.
		*N*, men: 28			
		Mean age (*SD*): 77.3 (7.2)			

*Note:* AD = Alzheimer’s disease; AS = Alzheimer’s Society; IPA = interpretive phenomenological analysis; MCI = mild cognitive impairment; MMI = mild memory impairment; NA = not applicable; NR = not reported; PWMCI = people with mild cognitive impairment; SD = standard deviation.

^a^Data generated with partners and PWMCI.

^b^Data generated with partners only.

^c^10 participants included in the analysis.

### Synthesis Findings

Two overarching conceptual themes were developed. The first, MCI and myself-in-time, examines the changes a MCI diagnosis brings with respect to the relationship between the self and the experience of time. Drawing on research from social psychology and phenomenological philosophy, the self is understood here as the locus of meaningful human agency and action (Merleau-Ponty, 1996). We emphasise this agentic self as being embedded in social relations, in cultural discourses—including discourses of aging and cognitive health (Allen, 2015; Gergen et al., 1996), and in time and place (Gomersall & Madill, 2014). The second, interlinked theme, living with ambiguity, describes difficulties in understanding the meaning of the diagnosis and its prognostic implications. Table 3 provides a sample of quotations illustrating the themes, concepts, or metaphors extracted from the individual studies, and indicates how these were linked to develop higher-order conceptual themes.

**Table 3.** Themes/Metaphors/Concepts Extracted From Primary Studies and Their Relation to the Synthesised Themes

Authors, year	Original theme/concept/metaphor extracted from study (with illustrative quotes)	Informed which metasynthesis theme(s)?
Banningh and colleagues (2008)	Loss of mastery: “having to abandon demanding activities was a recurrent theme”	The present: using it, losing it, and living for the moment
	Foreshadowed time: “Will I become demented”; “when I visit my sister in the nursing home I get upset and think: I’ll end up here too”; “he calls it forgetfulness, I call it the beginning of dementia”	The future: anxiety, open time, and fading time
	Normalising: “I’ve always been very absent-minded”; “when I observe that other people tell things twice, I think: So I’m not alone”; “many people of my age have this”	“Normal,” “non-normal,” or “pathological”?
	Problem-focused coping: “I make notes”; “I started doing crosswords and playing memory games”; I take asprin and I think that helps”	The present: using it, losing it, and living for the moment
Beard and Neary (2013)	Making sense of nonsense: My feeling is there is a lot of guesswork involved and that people don’t really know. Do you have early stage Alzheimer’s? Do you have MCI? Is there a difference? [It’s] a gray area. It’s like trying to make sense of nonsense.	Making sense of nonsense: Uncertainty in diagnosis and information
	When does it become a problem: If it gets to the point where I cannot do a straight tax and I don’t know where to begin with a client, then there’s something wrong	“Normal,” “non-normal,” or “pathological”?
	Dementia-related anxiety: What was unanimously and most commonly expressed, however, was the fear associated with Alzheimer’s—the “death sentence” diagnosis—and their determination not to “get it”	The future: Anxiety, open time, and fading time.
	Alzheimer’s-related stigma: some respondents reflected a lay understanding of what Goffman might have called a courtesy stigma potentially resulting from associating MCI with Alzheimer’s	“Normal,” “non-normal,” or “pathological”?
Berg and colleagues (2013)	When does it become a problem? “Difficulties in determining when a memory problem is normal and when it is related to a decline in cognitive health were expressed […] ‘but my wife forgets things too. So you know, it is like it depends on who it is that forget things. When does it become a problem?’”	“Normal,” “non-normal,” or “pathological”?
	Coping adaptations: “Coping mostly involved learning to know and to live in accordance with one’s own capabilities and how to avoid stressful situations”	The present: Using it, losing it, and living for the moment
	Future-oriented anxiety: “Participants expressed concern about not being able to continue performing activities in the future that were important in the present. ‘It’s about the capacity, you know, it gets poorer as time passes. And what I worry most about is that my memory will get worse, that I have to give up dancing. That I will forget the steps’.”	The future: Anxiety, open time, and fading time.
Blieszner and colleagues (2007) ^a^	Beyond “normality”: “Like Saide, many other spouses sought a medical explanation when they could no longer assume the forgetting was normal”	“Normal,” “non-normal,” or “pathological”?
	Supporting the self: “When providing reminders and supporting the elders, spouses also noted the value of maintaining the elders’ dignity and sense of self. This ability to think sensitively about the elders’ needs in the face of their own relational disruption is another example of resilience.”	The present: using it, losing it, and living for the moment
	Future-oriented uncertainty: “we planned on taking care of each other for as long as we could and outside that I don’t know”	The future: anxiety, open time, and fading time
	Problem-based coping: “Rather than withdrawing from social involvement, many couples relied heavily on calendars and note taking to keep up with medical appointments and social engagements and to record important information.	The present: using it, losing it, and living for the moment
Blieszner and Roberto (2010)	Switching roles: I take her someplace like Applebey’s and she seems kind of lost, and says, “What do you think I should have?” It’s very much like the roles are switched. And so I am ordering for her and doing for her what she would have done for me a long time ago.	The present: using it, losing it, and living for the moment
		The past: staying connected with the self
	Future-oriented anxiety: I don’t know what is going to be the worst the first, the mind or the physical body, because [husband] has three problems. He has got sugar, he has got [problems with] walking, and he has got a memory thing.	The future: anxiety, open time, and fading time
	Losing control: he won’t cooperate. And then, I get very, very angry. And, I don’t like that, because that has never been a part of me. So, that has been my biggest problem. I guess it’s because, all of my life, things have been in control. And, now they are not.	The present: using it, losing it, and living for the moment
		The past: staying connected with the self
Corner and Bond (2006)	Boundaries between “normal aging” and dementia: I was told I had some sort of dementia, but we weren’t sure whether that was different or normal for someone my age. That wasn’t made clear. We’re still not clear about what will happen. I mean I feel well, I don’t feel any different to what I did when I went first (to the family doctor). (Rose, 65, early-stage dementia) They said it was “cognitive impairment,” but that it was mild and we took comfort from that, I suppose. (Ron, 66, husband and caregiver to Rose)	“Normal,” “non-normal,” or “pathological”?
	Future-oriented anxiety: you picture these people who are vegetables . . . it’s horrific	The future: anxiety, open time, and fading time
	Problem-focused coping: they had developed coping strategies to deal with any memory lapses. For example, they had a specific place to put all keys, notes around the house, and written instructions on how to set the video	The present: using it, losing it, and living for the moment
	Uncertainty in diagnosis and information: To be fair, every time we go to see anyone about this, we feel more muddled rather than being put straight; we come away with more questions.	Making sense of nonsense: uncertainty in diagnosis and information
	Downward trajectory: Participants’ responses to people with dementia and perceptions of the experience of dementia were negative; a loss of independence, control, identity, and dignity were perceived to be inevitable.	The future: anxiety, open time, and fading time
Damianakis and colleagues (2010) ^a^	Connecting with past identity: Ms. K noted that this introspection had a positive influence on her sense of self: “I remember speaking to [social worker] after [the viewing] was over … I told her that I felt better about myself … because I saw a lot of good things in it … that I had allowed [myself] to be and to do.”	The past: staying connected with the self
	Nostalgia: “These are all people that I cared about, and they are no longer in existence … even if there’s some laughs in the movie I’m left with a little feeling of loss but it’s balanced to some extent by having a chance to see them again” (Mr. A, MCI, 81 years).	The past: staying connected with the self
	Fading time: “Not everybody has all the pictures … I was lucky to save all that … and to show it to my family and it’s going to stay with them … after I’m gone”	The future: anxiety, open time, and fading time
Davies and colleagues (2010) ^b^	Relational changes: In general, dementia group respondents expressed the fact that bilateral communication was nearly absent, whereas spousal caregivers of persons with MMI reported that they continued attempting to engage with their partners, and sometimes modified previous communication patterns (e.g., complexity or length) to facilitate understanding and satisfaction	The present: using it, losing it, and living for the moment
	Future-oriented anxiety, and living for the moment: “Participants in the MMI group expressed that they were handling the ambiguity of the future by remaining focused on the relationship in the present.	The present: using it, losing it, and living for the moment
	One male participant noted that he had heavily focused on researching ways to delay the disease progression in his partner. Another participant stated that one should be “grateful for what you have left and realize that you won’t get back what you had.”	The future: Anxiety, open time, and fading time.
Frank and colleagues (2006)	When does it become a problem? “Participants and informants discussed how memory and cognitive symptoms deviated from expectations about normal aging. Patients in both groups reported memory problems such as misplacing objects, word-/name-finding problems, and getting lost. Repetitive speech was also common to both diagnostic groups: “she does like to repeat stories and doesn’t realize she’s already told them” (MCI informant).	“Normal,” “non-normal,” or “pathological”?
	Uncertainty of diagnosis: “There was a degree of confusion around diagnosis in patient and informant groups, but participants accepted they may have forgotten what had been said. Many MCI patients had been told they had “mild memory loss” or “a memory problem that was not too bad,” but most were not given a specific name for their disorder. Attributions varied widely and several MCI patients described concern about developing AD. This uncertainty over diagnosis was corroborated by MCI informants: “not memory . . .it’s old age,” and “[it’s not] Alzheimer’s . . .it’s just that . . .the memory isn’t there.”	Making sense of nonsense: uncertainty in diagnosis and information
	Losing abilities: “The MCI patients all recognized that their current level of functioning was significantly worse than before. Many patients described frustration at not being able to do things as they used to. Reading, visiting friends (due to fear of getting lost and fear of not following conversations), hobbies, and work activities often suffered.”	The present: using it, losing it, and living for the moment
Kuo and Shyu (2010) ^b^	Ambivalent normalization: “Participant 3 said, ‘She always forgot the place where she put her articles for daily use. That seemed like a *normal phenomenon* because I am also forgetful.’” (emphasis added)	“Normal,” “non-normal,” or “pathological”?
	Subtle changes: “Some of the elders’ behavioural and personality changes were so slight and seldom occurred that the family caregivers were at first not aware of the changes”	“Normal,” “non-normal,” or “pathological”?
Lingler and colleagues (2006)	Uncertainty in diagnosis: In response to our broad introductory question […], only 1 respondent explicitly invoked the clinical label MCI […] upon clarification by the interviewer, 10 of the 11 other participants affirmed that they had been formally diagnosed	Making sense of nonsense: uncertainty in diagnosis and information
	Relief: Examination of the narrative accounts revealed that feelings of relief typically emerged within the context of a looming Alzheimer’s disease diagnosis.	“Normal,” “non-normal,” or “pathological”?
	“Normal,” “non-normal,” or “pathological”? Half of those who positively framed their emotional appraisals [of MCI…] perceived mild cognitive impairment to be an expected aspect of the aging process. Distressed appraisers, in contrast, did not engage in such normalizations.	“Normal,” “non-normal,” or “pathological”?
	Future-oriented anxiety: ‘‘MCI to me means that it would lead to AD’’ […] References to uncertainty and apprehension regarding the future were frequently embedded within prognosis-focused appraisals	The future: anxiety, open time, and fading time
	Enjoying the moment: just the realization that we’re getting older ...I savor the things that are all around us. I enjoy them. I enjoy seeing the sun come up, and go down when I go to bed. And, I watch the moon a lot ... I wish I could just slow things down.	The present: using it, losing it, and living for the moment
Lu and Haase (2009) ^b^	Losing capabilities: “Caregivers realized that their spouses were having difficulty engaging in previously familiar tasks such as putting household items away, changing light bulbs, preparing dinner, or using “the to-do list” as usual.”	“Normal,” “non-normal,” or “pathological”?
	Future-oriented anxiety: “Matthew: “I really dread the day when she won’t be able to have her checkbook…when she won’t be able to drive… because I know how she’s going to react and she’s going to be very bitter, very bitter”;”	The future: Anxiety, open time, and fading time
	When is it a problem? “All caregivers experienced difficulty in sorting out the differences between the signs of memory decline, normal aging, and working stress”	“Normal,” “non-normal,” or “pathological”?
	Fading time (and space): “Caregivers also experienced a shrinking world, including shrinking spaces, activities, finances, and social relationships. The aspects of the caregivers’ world that were shrinking included moving to more manageable housing, shifting to communities that were unfamiliar, compacting life routine, dwindling financial resources, and devastating relationship changes.”	The future: anxiety, open time, and fading time
	Affective impact: “they [caregivers] intermittently experienced unpredictable periods of heightened emotional distress as they observed the consequences of their spouse’s functional decline. The range of distressful emotions was linked to the situations and included shock, anger, guilt, anxiety, frustration, sadness, loneliness, helplessness, worry, and uncertainty.”	The present: using it, losing it, and living for the moment
Roberto and colleagues (2013) ^a^	Active coping: “I just accept it as part of the natural aging process. I am trying to, for example, I am doing crossword puzzles and jigsaw puzzles, and I am trying to learn to play bridge better. That is a hopeless task I think, but I volunteer. I work at the county library one day a week”	The present: using it, losing it, and living for the moment
	Changing relationships: Other couples shared responsibilities throughout their marriage, but with the progression of MCI, the care partner took on more and more responsibilities that the PWMCI once held.	The present: using it, loising it, and living for the moment
Lingler and colleagues (2006)	Uncertainty in diagnosis: In response to our broad introductory question […], only 1 respondent explicitly invoked the clinical label MCI […] upon clarification by the interviewer, 10 of the 11 other participants affirmed that they had been formally diagnosed	Making sense of nonsense: uncertainty in diagnosis and information
	Relief: Examination of the narrative accounts revealed that feelings of relief typically emerged within the context of a looming Alzheimer’s disease diagnosis.	“Normal,” “non-normal,” or “pathological”?
	“Normal,” “non-normal,” or “pathological”? Half of those who positively framed their emotional appraisals [of MCI…] perceived mild cognitive impairment to be an expected aspect of the aging process. Distressed appraisers, in contrast, did not engage in such normalizations.	“Normal,” “non-normal,” or “pathological”?
	Future-oriented anxiety: ‘‘MCI to me means that it would lead to AD’’ […] References to uncertainty and apprehension regarding the future were frequently embedded within prognosis-focused appraisals	The future: anxiety, open time, and fading time
	Enjoying the moment: just the realization that we’re getting older ...I savor the things that are all around us. I enjoy them. I enjoy seeing the sun come up, and go down when I go to bed. And, I watch the moon a lot ... I wish I could just slow things down.	The present: using it, losing it, and living for the moment
Lu and Haase (2009) ^b^	Losing capabilities: “Caregivers realized that their spouses were having difficulty engaging in previously familiar tasks such as putting household items away, changing light bulbs, preparing dinner, or using “the to-do list” as usual.”	“Normal,” “non-normal,” or “pathological”?
	Future-oriented anxiety: “Matthew: “I really dread the day when she won’t be able to have her checkbook…when she won’t be able to drive… because I know how she’s going to react and she’s going to be very bitter, very bitter”;”	The future: Anxiety, open time, and fading time
	When is it a problem? “All caregivers experienced difficulty in sorting out the differences between the signs of memory decline, normal aging, and working stress”	“Normal,” “non-normal,” or “pathological”?
	Fading time (and space): “Caregivers also experienced a shrinking world, including shrinking spaces, activities, finances, and social relationships. The aspects of the caregivers’ world that were shrinking included moving to more manageable housing, shifting to communities that were unfamiliar, compacting life routine, dwindling financial resources, and devastating relationship changes.”	The future: anxiety, open time, and fading time
	Affective impact: “they [caregivers] intermittently experienced unpredictable periods of heightened emotional distress as they observed the consequences of their spouse’s functional decline. The range of distressful emotions was linked to the situations and included shock, anger, guilt, anxiety, frustration, sadness, loneliness, helplessness, worry, and uncertainty.”	The present: using it, losing it, and living for the moment
Roberto and colleagues (2013) ^a^	Active coping: “I just accept it as part of the natural aging process. I am trying to, for example, I am doing crossword puzzles and jigsaw puzzles, and I am trying to learn to play bridge better. That is a hopeless task I think, but I volunteer. I work at the county library one day a week”	The present: using it, losing it, and living for the moment
	Changing relationships: Other couples shared responsibilities throughout their marriage, but with the progression of MCI, the care partner took on more and more responsibilities that the PWMCI once held.	The present: using it, loising it, and living for the moment
Roberto and colleagues (2011)	MCI acceptance: We identified four degrees of acknowledgment of MCI within the family members’ interviews: complete acknowledgment, passive acknowledgment, partial acknowledgment, and no acknowledgment. […] the four groups [are] situated on a continuum from complete acknowledgment to complete denial.	“Normal,” “non-normal,” or “pathological”?
	Importance of knowledge and assumptions: families with a person working in a health care field tended to handle the problems associated with MCI matter-of-factly and did not necessarily equate MCI with AD or advanced dementia.	“Normal,” “non-normal,” or “pathological”?
	Family influences: “Patterns related to family-level power dynamics varied among the four degrees of acknowledgment. Sixty-seven percent of complete acknowledgers reported that the PCP (or occasionally the SCP) had always been in charge in the family […] All families characterized by passive acknowledgment reported family dynamics where the PCP or SCP took charge because of deteriorating physical health”	The present: using it, losing it, and living for the moment
	Coping, social support, and their limits: Coping strategies such as making reminder lists were used frequently by elders in all but the passive acknowledgment group […] It is important to note, however, that the elders’ use of strategies such as keeping notes or elaborate calendars had limited effectiveness if others in their family did not acknowledge the extent of the elder’s memory problems	The present: using it, losing it, and living for the moment
		The past: staying connected with the self
Roberts and Clare (2013)	Maintaining independence: for those living alone, retaining their sense of independence appeared extremely important, especially in light of changes to memory and the possible perceived risk of that independence being taken away.	The present: using it, losing it, and living for the moment
	Social support: there was a man and I knew the way he was looking at me that he knew me. I couldn’t work out who he was so I asked (wife) quietly	The present: using it, losing it, and living for the moment
	Foreclosed time: His way of dealing with it (husband) is to ignore it completely, he doesn’t want to know but you see he only knows about Alzheimer’s from people in the latter stages . . . and I think that frightens him	The future: anxiety, open time, and fading time
	“Normal,” “non-normal,” or “pathological” aging? “interview extracts reflected a sense that life went on as normal, regardless of age, memory clinic attendance or perceived changes in memory.	“Normal,” “non-normal,” or “pathological”?
	Lack of information: In some instances, direct reference was made to the lack of information supplied at the time of the memory clinic assessment. Shirley blamed herself for not asking what the outcome of assessment could mean for her.	Making sense of nonsense: uncertainty in diagnosis and information
	Coping with humour: humour was used by some interviewees to divert the conversation from serious, possibly upsetting occurrences in participants’ lives	The present: using it, losing it, and living for the moment
	Absence of a label: throughout the interviews the term MCI was not adopted by any of the participants. Whether or not this reflects the level of information provided at diagnosis, or a lack of knowledge surrounding the MCI term, the absence of a label seemed to increase the uncertainty.	Making sense of nonsense: uncertainty in diagnosis and information
	Stigma: . . . because a lot of people put two, two together say, oh he’s going round the bend he is, you what I mean (laughs) you know what I mean so people so I-I-I just don’t go down that road no more	“Normal,” “non-normal,” or “pathological”?
	Losing the self: participants also felt that they were different people and that they could not be relied upon in the way that they once were. Rather than seeing themselves as having memory difficulties resulting in unreliability, they saw themselves as unreliable	The present: using it, losing it, and living for the moment
		The past: staying connected with the self
Rosenberg and Nygård (2013)	Learning as doing: The interplay between continuously using everyday technology and maintaining or achieving new knowledge of how to use everyday technology was understood as creating an intertwined process where the doing was most important […] However, the drilling effect was only temporary; even short periods of not using an everyday technology, or certain functions in a piece of technology, easily led to losing their know-how, necessitating relearning.	The present: using it, losing it, and living for the moment
	‘Preventive’ and ‘momentary’ learning: Preventive management strategies were set up beforehand by the person, while momentary strategies appeared while using the technology […]The most common preventive management strategy was using written notes […]The most common [momentary] strategy […] was to approach problems by trial and error as they occurred.	The present: using it, losing it, and living for the moment
	Social support in learning: could (a) motivate participants’ technology use, (b) provide them with technology, and (c) force them to use technology. Also, other persons could (d) support participants in using technology through teaching them, and (e) give practical support to solve problems related to technology use.	The present: using it, losing it, and living for the moment
	Levels of self-technology interaction: On the first level, the participants waited for signals from the technology, on the second level they gave single commands to the technology, and on the third level they interacted with the technology in longer sequences of actions and responses.	The present: using it, losing it, and living for the moment
Saunders and colleagues (2011)	Face saving: “From the perspective of the person with CI, the doctor’s office may represent a face-threatening situation […] He or she may be framed as deficient or somehow socially unacceptable when failing to perform at memory tasks and orientation assessment	“Normal,” “non-normal,” or “pathological”?
	Communicative coping behaviours: memory accounts; health accounts, humor	“Normal,” “non-normal,” or “pathological”?

^a^Fieldwork carried out with partners of people with MCI only.

^b^Fieldwork carried out with people with MCI and their partners.

## Theme 1. MCI and Myself-in-Time

### Subthemes

#### The past: Staying connected with the self

It is well-known that the onset of neurocognitive illness can mark a profound re-evaluation of one’s past. As a person comes to terms with a new position in the world, their biography needs rethinking, and many participants throughout the literature spoke of how they had changed relative to the past (e.g., Banningh, Vernooij-Dassen, Rikkert, & Teunisse, 2008; Davies et al., 2010; Lu & Haase, 2009). Damianakis and colleagues (2010) was highly past-focused, and described some nuanced ways in which people with MCI related to this temporal mode. These investigators developed multimedia biographies—DVDs comprising photographs, film footage, and other archival material from participants’ lives—to capture “a historical perspective of interactive communication with family and friends” (p. 25). The authors reported a positive response to the biographies, arguing that they promoted continuation of memory and the self; as one participant put it, they “enriched the memories [she] already had” (p. 29). Others reported vicarious enjoyment as they saw themselves in times and places they had once known: “I was […] almost able to look down that lane way and see that lovely part of the bush I used to go” (p. 29). This same participant also noted how the biography promoted a positive sense of self, because she “saw a lot of good things in it … that I had allowed [myself] to be and do” (p. 29). Despite the generally optimistic tenor of participants’ responses to the multimedia biographies, some accounts were infused with multiple, contrasting affective valences—humour, enjoyment, sadness, nostalgia, and loss:

These are all people that I cared about, and they are no longer in existence … even if there’s some laughs in the movie I’m left with a little feeling of loss but it’s balanced to some extent by having a chance to see them again (pp. 29–30)

Nostalgia for past relationships, roles, and experiences was widespread through the literature, and the only study in which this phenomenon was absent was Berg, Wallin, Nordlund, and Johansson (2013). In that article, participants described anxiety in the past, and contrasted this with a more hopeful, optimistic present. This difference was likely related to the characteristics of these participants: they had been diagnosed seven or more years ago, but their cognitive impairments had not progressed, and they were consequently less worried about the possibility of dementia in the future. It follows that the nature and progress of cognitive impairments profoundly influences one’s experience of temporality. A new, uncertain diagnosis may lead to a sense of open time and/ or future-oriented anxiety, whereas progressive decline implies fading time; conversely, stable or improving cognitive abilities can lead to restorative time. As our analysis has shown, the way in which one interprets MCI in terms of temporality carries important implications for well-being.

#### The present: Using it, losing it, and living for the moment.

Participant narratives of lost capabilities were heterogeneous, but widespread. Some spoke of giving up work or similar responsibilities (e.g., “I ceased doing administration for the association,” Banningh et al., 2008, p. 151), or other constraints on the self in everyday life (“I used to be quite proud of my vocabulary but now I really find it hard to express myself the way I want,” Frank et al., 2006, p. 157). Some researchers, such as Beard and Neary (2013), while noting declining cognitive abilities among their participants, couched these difficulties as “obstacle[s] to overcome” (p. 137)—highlighting the continued role of agency in participants’ lives, their sense of open time, and resistance toward deterministic narratives of decline and incapacity. Indeed, various psychological coping approaches were discussed (Banningh et al., 2008; Beard & Neary, 2013; Blieszner, Roberto, Wilcox, Barham, & Winston, 2007; Davies et al., 2010; Kuo & Shyu, 2010; Roberto, Blieszner, McCann, & McPherson, 2011; Roberts & Clare, 2013; Roberto, McCann, & Blieszner, 2013). Others construed the present as a tragic realm of fading capabilities, in which further decline was profound and inevitable. Lu and Haase (2009), for example, describe “a shrinking world, including shrinking spaces, activities, finances, and social relationships” (p. 388). The participants in this study struggled to find ways to live well with MCI, and a range of distressing emotions associated with their loved ones’ functional decline were reported, including “shock, anger, guilt, anxiety, frustration, sadness, loneliness, helplessness, worry, and uncertainty” (p. 389).

Arguably, such despairing affect resulted from a broader temporal perspective—for example, comparing a damaged present self with an idealised past, masterful self, or extrapolating losses into the future. Conversely, “living for the moment” was sometimes drawn on by people with MCI to find continued enjoyment and meaning. Davies and colleagues (2010) noted their participants “handled the ambiguity of the future by remaining focused on the relationship in the present” (p. 623), with one participant arguing that one should be “grateful for what you have left and realise that you won’t get back what you had” (ibid). Others mentioned the valued activities they were able to continue (e.g., “I’m still being asked for the choir,” Banningh et al., 2008, p. 152).

Perhaps the most present-focused study was Rosenberg and Nygård (2013). The authors viewed participants’ engagement with everyday technology as an “intertwined process” of learning and doing (p. 667), and described “preventive” and “momentary” strategies of self-management. Preventive strategies involved explicit forward-planning, for example, using written instructions or manuals. However, participants often found these cerebral approaches difficult, and expressed a preference for momentary strategies—learning by doing. Drawing attention to the importance of embodiment in maintaining activity, this process of trial and error was conceptualized “as a conscious way to give the doing a flow and allow the participant access to his or her unreflected and automated knowledge, when the body knows how to act without thinking” (p. 669). Hence, a present-oriented temporal perspective may help people with MCI on two levels. With respect to emotion, being-in the present may mitigate the sense of anxiety associated with future-orientation, or the sense of loss that is sometimes associated with past-orientation. Second, with respect to everyday activity, the capacity to immerse oneself in the doing may be one route to develop and maintain mastery.

At times, participants’ accounts combined a variety of temporalities. One participant in the Lingler and colleagues (2006) study was an exemplar, expressing an interlinked experience of present- and future-oriented temporalities: living for the moment and fading time:

…Just the realization that we’re getting older … I savor the things that are all around us. I enjoy them. I enjoy seeing the sun come up, and go down when I go to bed. And, I watch the moon a lot … I wish I could just slow things down (p. 797)

#### The future: Anxiety, open time, and fading time

Anxiety and worries about the future were ubiquitous through the literature. Many participants perceived MCI as an early or mild form of dementia, anticipating an inevitable decline in agency and selfhood: “We’ve just been turned upside down by it…. You picture these people who are vegetables … it’s horrific” (Corner & Bond, 2006, p. 8). “When I visit my sister in the nursing home I get upset and think: I’ll end up here too” (Banningh et al., 2008, p. 151). Such examples suggest what Morson (1996) calls “foreshadowing”: a temporality in which the future has already been decided, thereby removing possibility and agency from the present. Reflecting widespread narratives of dementia representing a devastating illness that progressively robs people of their dignity and autonomy, thoughts of the future were often characterized by anguish, fear, and hopelessness. The Lu and Haase (2009) article was especially striking in this regard: one of the key concepts developed in their study comprised “a downward spiral into a world of silence” (p. 388).

It was not only the perception of MCI as a prodromal form of dementia that led to future-oriented anxiety; concerns were also raised around the uncertainty of the label (“This is the beginning, but where will it end?” Banningh et al., 2008, p. 152; see also Beard & Neary, 2013; Davies et al., 2010; Lu & Haase, 2009). A participant in Lingler and colleagues (2006) interpreted MCI to imply: “a problem and I don’t … know whether it… will continue to generate or whether it can be turned around” (p. 796). Another individual in that study also proffered a vision of open time with respect to the future, refusing to discount possibilities of future improvement, decline, or stasis: “maybe I can get improvement […] Hopefully it’s going to get worse, not hopefully, but possibly it will” (ibid). In these accounts, there was a sense of time remaining open, not (yet) subject to the loss narratives often presupposed of dementia.

At other times, participants expressed their sense of fading time; the notion that most of life’s possibilities were behind them. It was not always clear whether this was due to participants’ understanding that they were heading for the end of life, or if it had to do with awareness of the possible degenerative nature of neurocognitive impairments. However, the expression of fading time was not always as negative as one might expect: “The future? Oh, the future is behind me! (laughs) you know, I have had a great life, I have a good job and kids and… all that is behind me you know. I just hope I will have some good and healthy years now” (Berg et al. 2013, p. 297). Here, the future is construed as peripheral—the important thing was to reflect back on a life well-lived (Damianakis et al. 2010).

## Theme 2: Living With Ambiguity

### Subthemes

#### Making sense of nonsense

Uncertainty in diagnosis and information. Although a considerable body of research is beginning to investigate ways to reliably identify MCI using neurological, psychological, and even genetic tests, the extent to which MCI can be distinguished in routine practice is uncertain. Indeed, the literature showed some people with MCI explicitly questioning the clinical utility of the label. As one reflected: “My feeling is there is a lot of guesswork involved and that people don’t really know. Do you have early-stage Alzheimer’s? Do you have MCI? Is there a difference? […] It’s like trying to make sense of nonsense” (Beard & Neary, 2013, p. 138). In other studies, MCI was seen as ruling out dementia (Lingler et al., 2006), or as a precursor thereof (Banningh et al., 2008; Frank et al., 2006). Roberto and colleagues (2011) highlighted a variety of interpretations people made of the label.

Perhaps one reason for this uncertainty was heterogeneity in the way participants were diagnosed. In Lingler and colleagues (2006), only one of 11 participants explicitly invoked the term MCI when asked about diagnosis. They elaborate: “upon clarification by the interviewer, 10 of the 11 other participants […] affirmed that they had been formally diagnosed with [MCI…] One individual stated that she did not recall ever hearing the term, explaining that she believed that she had been diagnosed with mild Alzheimer’s disease. Hence, we excluded her data” (p. 795). The contrast of this decision with the approaches described in other studies is striking. Corner and Bond (2006), for instance, described the difficulties involved in recruiting people with MCI, and so opted to include people with early-stage dementia. Other participants reported being explicitly told they had dementia (Blieszner et al., 2007), or “had been told they had ‘mild memory loss’ or ‘a memory problem that was not too bad,’ but most were not given a specific name for their disorder” (Frank et al., 2006, p. 156). Nevertheless, some participants reported that having a label helped them make sense of their cognitive difficulties (Lingler et al., 2006)—and expressed relief that they had not been diagnosed with Alzheimer’s disease (Lingler et al., 2006). Such variation and uncertainty in the labelling of MCI thus seems to be widespread among clinicians and researchers; a phenomenon which was reflected in the accounts of people with MCI themselves.

However, what actually happened in encounters between clinicians and people with MCI was unclear. As Banningh and colleagues (2008) noted, “[we did not] monitor the effect of the consultation in which the geriatrician disclosed and explained the MCI diagnosis […] besides MCI, clinicians use numerous other labels” (p. 153). Although these authors go on to make a plea for “the provision of accurate and current information about MCI, separating fact from fiction” (p. 153), it remains uncertain whether fact and fiction have yet been distinguished even in the expert discourses of research and medicine. Difficulties people with MCI experienced in obtaining helpful information—both in terms of diagnosis and ongoing support—were widespread (Beard & Neary, 2013; Blieszner et al., 2007; Frank et al., 2006; Lu & Haase, 2009; Roberts & Clare, 2013). “Forced to seek information on their own, some spouses turned to the internet, public library, and occasional brochures they found at doctors’ offices […] But in general, the majority found very little material specific to MCI and written for lay audiences” (Blieszner et al., 2007, p. 200). The lack of information and conceptual clarity about MCI led to a pervasive subjective sense of ambiguity.

#### “Normal,” “non-normal,” or “pathological?”

Linked with the uncertain status of MCI as a diagnostic entity, many research participants had questions concerning the extent to which MCI represented a “real” problem. Beard and Neary (2013) reported widespread resistance among participants to the medicalization of their cognitive capacities. They insisted that MCI was part and parcel of getting older, and explicitly rejected the possibility of dementia. Notably, in this study, “many were not sure that [MCI] had ever been defined for them” (p. 138). Another example of normalising was seen in Banningh and colleagues (2008). Participants attributed MCI to external factors, or again, explicitly said it wasn’t dementia. Roberto and colleagues (2011) noted differing levels of acceptance of MCI among participants. One subgroup, which these researchers call “partial acknowledgers,” “tended to reveal some awareness that the elder had memory or cognitive difficulties, but not all members indicated that they accepted it as a legitimate medical condition over which the elder had no control” (pp. 761–762). However, they also described groups of participants who entirely rejected, or entirely accepted, the medical validity of MCI. Clearly, the relationship between MCI, dementia, and “normal” aging was difficult for people with MCI to unpick in the process of sense-making.

As hinted by these varied accounts, the term “normal” carries two distinct but linked meanings: normative, and nonpathological. From a social constructionist perspective, Saunders and colleagues (2011) noted that people with AD or MCI often accounted for memory lapses by referring to non-AD reasons. They see this as a protection of “face,” because of the social stigma associated with AD. It is possible that widespread normalizing of MCI may, in part, be attributable to the desire to project a valued, normative social identity (Beard & Neary, 2013, p. 139). Indeed, dementia was often viewed as bearing tragic existential connotations: “It’s a death sentence … Means loss of function [….] as a human being. A loss of capacity to be one’s self […] the worst of all insults” (Beard & Neary, 2013, p. 140). At other times, however, MCI was seen as precluding dementia. This provided relief on two levels: first, in terms of ruling out dementia, and second, by providing a diagnostic label through which people could better make sense of the problems they had been experiencing:

Well, to be quite honest, I think I was relieved […] I was concerned probably like everyone else would [be], that I had Alzheimer’s, and then he said, ‘you don’t have Alzheimer’s,’ and you know that’s like taking a cloud off your shoulder […] so giving it a title, you can call it anything you want, but it’s not Alzheimer’s, so I can live with it (Lingler et al., 2006, pp. 795–796)

## Discussion

This metasynthesis examined the qualitative literature pertaining to the experience of being diagnosed, and living with, MCI. The first theme, MCI and myself-in-time, showed the various ways in which the diagnosis and effects of MCI could impact one’s sense of being-in time. The second theme, living with ambiguity, underlined the difficulties people have in making sense of MCI, and the clinical and social implications it might have for them. There is wide variation in the way diagnosis is delivered, and the meaning of “normal aging” in relation to MCI is ambiguous, because it carries both sociological (i.e., normative) and medical (i.e., nonpathological) meanings (see Misztal, 2001, for an interesting discussion of normality in social theory). This, in turn, reveals an ethical tension between providing the best early interventions for people with MCI on the one hand, and potentially stigmatising older people on the other. Temporality and the multiplicity of possible MCI meanings are closely interlinked, too, with the wide interpretive scope within a MCI diagnosis permitting a range of temporal implications. For instance, interpreting MCI to signify early dementia may lead one to feel subject to linear narratives of decline and debility; conversely, viewing MCI as an open-ended label which may or may not imply incipient dementia could promote an open-ended temporality.

There are important interrelationships between the themes of this metasynthesis: When a MCI diagnosis is delivered, depending on the nature of the clinical communication and the person’s perspective on the label, MCI can be interpreted in one of three ways: as (a) “normal,” (b) “non-normal,” or (c) “pathological.” These three interpretations, in turn, each bear a distinct set of implications for the temporal being of the self. We are not suggesting these interpretations act upon the self in a deterministic way; but rather that they help circumscribe the range of possibilities a person can envisage for herself. Indeed, our findings were suggestive that other mediating factors, including coping styles, level of social support, the experience of clinical assessment and diagnosis, and severity of cognitive difficulties, also appear to play into the relationship between MCI and the temporal situatedness of the self. It would perhaps be productive to examine the relationships between such constructs more systematically in future qualitative and quantitative studies.

There are several limitations to our findings. Perhaps most importantly, the ambiguity in MCI fed into the metasynthesis, such that we could not tell if we were comparing accounts from people with similar cognitive issues. Some studies included participants with AD (e.g., Corner & Bond, 2006; Davies et al., 2010; Damianakis et al., 2010; Rosenberg & Nygård, 2013), and slippage between “MCI” and “early-stage AD” could be seen in several papers. Davies and colleagues (2010) consider that MCI and memory impairment “may represent prodromal forms of dementia” (p. 618), whereas Blieszner and Roberto (2010) claim that their study “extends previous work on ambiguous loss associated with AD to an earlier stage of cognitive decline” (p. 206). Although this creates some interpretive difficulties, it could be seen as a pertinent reflection of the nature of MCI itself as an ambiguous phenomenon.

Second, it is important to recognise the “third-order” interpretations on which our analysis was based (Malpass et al. 2009, p. 158; see also the Methods section in the present article). Although this is not a limitation per se—and is, arguably, a strength of interpretive reviews (Zimmer, 2006)—it is nevertheless important to employ some reflexivity, and be clear about how the synthesis was impacted by the assumptions we brought to the analysis. The use of temporality as an analytical framework, for example, was undoubtedly influenced by the lead author’s pre-existing interest in this topic (references removed for blind peer review). Others in our group are interested in the linkages between technology and well-being (references removed for blind peer-review). Arguably, this meant that we placed more weight on research papers exploring these issues than scholars with differing interests might have done. Indeed, we would encourage others to explore the literature for themselves, and to test their interpretations against our own.

To conclude, we offer brief reflections on key ethical and psychosocial issues in MCI diagnosis, along with some implications for practice. First, disclosing a MCI diagnosis appears to be a double-edged sword. Benefits to patients might include intervening early, promoting self-management, and enabling the use of a medical label to make sense of one’s issues. By contrast, the ambiguity surrounding MCI may complicate such sense-making, and some have argued that the term may be stigmatising. This is an issue to which practitioners should remain sensitive. Allen (2015) has provided a helpful overview of research showing that ageism can have deleterious impacts, not only on subjective well-being, but also on the development and course of chronic illnesses. Hence, one fruitful avenue may involve emphasising the competencies of people with MCI to promote continued positive engagement in challenging social and cognitive activities that the person has reason to value. Indeed, evidence is emerging on the potential for such engagement to improve cognitive capabilities (e.g., Carlson et al., 2008; Tranter & Koutstaal, 2008). Werner and Korczyn (2008) make the important point that clinicians should be clear with patients that MCI does not necessarily lead to dementia, and it is important that patients understand their prognosis as well as possible. Hence, psychological research into risk communication may be an important avenue for future research in MCI. Explaining prognosis well is likely to be especially challenging for practitioners, given persisting diagnostic controversies. This also throws up questions about potential support for people with MCI. For example, how can psychosocial and other health care services remain attentive to the depth, complexity, variability, and significance of the way the syndrome plays out in individual lives? Although we do not claim to have satisfactory answers to such questions, our hope is to frame some potential avenues for further work in light of the metasynthesis findings.

Second, the potential trade-off between early support and stigmatization suggests an imperative to develop and evaluate services and technologies of specific relevance to MCI. There may be an opening, when a person is diagnosed, to develop early support and preventive practice to avoid unnecessary disability and ill-being caused by MCI. One possibility we are currently exploring, for example, concerns digital technology. Many digital devices are currently being developed to assist people with MCI in activities of daily living such as navigation, remembering appointments, and maintaining cognitive abilities. In addition to such disability mitigating tools, a small body of evidence is emerging to suggest using novel digital technology can enhance cognitive function (e.g., Chan, Haber, Drew, & Park, 2014), improve quality of life (Schulz et al., 2014), as well as being an enjoyable learning process in itself (Rosenberg & Nygård, 2013). In addition, our findings echo previous suggestions that supporting people with neurocognitive disorders such as dementia to place themselves in a meaningful temporal framework is fundamental to well-being (e.g., Nygård & Johansson, 2001). Again, new assistive technologies could play a role here—including “multimedia biographies” of the kind studies by Damianakis and colleagues (2010), or, more pragmatically, those that allow people to orient themselves in time and place (reminder systems, automated calendar reminders, etc.). However, the increasing uptake of digital technologies in research and practice can raise a new set of ethical challenges (e.g., Eccles, Damodaran, Olphert, Hardill, & Gilhooly, 2013; Greenhalgh, Stones, & Swinglehurst, 2014), and the evaluation science for such complex, socially situated systems often lags behind the rapid technology development. New evaluation approaches are therefore needed to analyse whether such solutions are helpful for people with MCI. In addition, technology is but one potential route for supporting people with MCI; we would argue that the important thing is to help people engage in activities that are meaningful to them personally—and this could include practices such as art, music, gardening, or involvement in local community groups.

## Funding

We are grateful to the Canadian Institutes of Health Research, the Economic and Social Research Council (UK), and the Swedish Council for Working Life and Social Research, for funding the Ambient Assisted Living for Wellness and Engagement in Later Life project (www.all-well.org) within which this review was undertaken.
